# Effects of in-car CO2 concentration on driving: a preliminary study with taxi drivers

**DOI:** 10.1539/eohp.2025-0001

**Published:** 2025-08-27

**Authors:** Kan Shimazaki, Yo Ishigaki, Kazunori Hayashi, Koji Fujita

**Affiliations:** 1 Faculty of Biology-oriented Science and Technology, Kindai University, Wakayama, Japan; 2 Research Center for Realizing Sustainable Societies, The University of Electro-Communications, Tokyo, Japan

**Keywords:** carbon dioxide, cognitive function, driving performance, driving simulator, in-car air quality

## Abstract

**Objectives:**

To evaluate the effects of carbon dioxide (CO2) concentrations in automobiles on driving performance.

**Methods:**

A driving simulator experiment was conducted with eight taxi drivers. The experiment was conducted under low CO2 concentration (<500 ppm) and high CO2 concentration (5,000 ppm) conditions. To evaluate driving performance and cognitive function, three measures were employed: a two-back task, an LED response task, and a driving assessment. The driving assessment used scoring criteria from the driving license proficiency test.

**Results:**

Poisson regression analysis showed that wobble (p=0.044), signal failure (p=0.045), contact (p=0.003), and wheel departure (p=0.005) were significantly increased under high CO2 concentration conditions. Generalized linear mixed model (GLMM) analysis showed that reaction time in the LED response task was significantly reduced under high CO2 concentration conditions (p<0.001). On the other hand, the GLMM analysis of the two-back task showed no significant effect of CO2 concentration (incorrect response rate: p=0.733, non-response rate: p=0.485).

**Conclusions:**

These results suggest that elevated CO2 concentrations may have a negative impact on driving behavior, especially skill-based driving behavior. On the other hand, the effects on cognitive tasks requiring working memory were limited. The results of this study suggest that managing CO2 concentration in vehicles is important for maintaining safe driving and raise the need for specific measures, such as the development of systems for measuring, predicting, and controlling CO2 concentration in vehicles, and the implementation of driver education programs.

## Introduction

Carbon dioxide (CO2) concentration serves as a key indicator of air quality in vehicles. Studies have demonstrated that CO2 concentrations frequently exceeded 4,000 ppm in taxis and buses^1)^ and surpassed 5,000 parts per million (ppm) in passenger cars. Research^2)^ revealed that when vehicles operated in the outside air introduction mode, CO2 concentrations remained relatively stable at around 1,000 ppm. However, in the inside air circulation mode, CO2 levels rose dramatically — reaching maximum concentrations of 4,520 ppm for highway driving, 4,730 ppm for suburban driving, and 6,770 ppm for urban driving^2)^. These variations in maximum CO2 concentrations across different driving environments may be attributed to differences in natural ventilation rates due to vehicle speed, external CO2 sources (traffic density, surrounding buildings), and occupant-related factors (passenger numbers, activity levels). Despite these variations, the data clearly demonstrate that the use of inside air circulation mode leads to CO2 concentrations that are an order of magnitude higher than outdoor atmospheric levels (approximately 400 ppm). These levels are considerably higher than those typically recommended for indoor environments^1)^.

Recent studies^3,4,5)^ have revealed that CO2 concentrations in the range of thousands of ppm, previously considered benign, can significantly impact human cognitive function. Studies demonstrated that at 1,000 ppm CO2 concentration, a significant reduction was observed in decision-making capacity^3)^. Research in simulated office environments^4)^ demonstrated a 21% decline in cognitive scores when CO2 concentrations rose from 550 ppm to 1,400 ppm, with pronounced effects in higher-order cognitive domains, including crisis response, information utilization, and strategic thinking.

A flight simulator experiment^5)^ with 30 commercial aircraft pilots demonstrated significant performance improvements at lower CO2 concentrations (700 ppm vs. 2,500 ppm; odds ratio 1.69; 95% confidence interval [CI], 1.11–2.55). Similarly, a focused study on driving contexts^6)^ using a driving simulator with 50 participants demonstrated that elevated in-vehicle CO2 levels (exceeding 1,400 ppm) correlated with increased traffic violation rates and driver fatigue levels. This evidence directly links CO2 concentration to driving performance and safety-related behaviors.

Building upon these previous findings, the primary objective of this study is to evaluate the effects of elevated CO2 concentrations in vehicle cabins on the driving performance and cognitive function of professional taxi drivers. We hypothesize that exposure to high CO2 levels (5,000 ppm) will result in: (1) decreased performance on cognitive tasks, particularly those requiring working memory and attention; (2) increased errors and longer reaction times in simulated driving tasks; and (3) a higher frequency of driving errors as assessed by professional driving instructors.

The study’s novelty lies in examining a realistic high-concentration CO2 environment (5,000 ppm) using taxi drivers as subjects, addressing a gap in previous research^6)^. While prior studies typically used CO2 concentrations of 1,000–2,000 ppm, this study employs a higher concentration to simulate extreme but plausible in-vehicle conditions. As a preliminary investigation, this study with a small sample of professional drivers aims to provide insights into CO2’s impact under realistic conditions, while allowing for detailed individual performance analysis and methodological refinement.

Our comprehensive approach combines assessment of driving behavior based on actual driving test scoring criteria with multiple cognitive tasks. By implementing a continuous driving task lasting approximately 1 hour, the study accounts for potential cumulative effects of prolonged CO2 exposure, mirroring real-world conditions more closely than shorter-duration experiments.

The significance of this study is underscored by the higher accident rates observed among taxi drivers in Japan. Studies have shown that the traffic accident rate per vehicle kilometer for taxis is more than 1.5 times higher than other vehicles^7)^, with accidents involving pedestrians and bicycles occurring at more than twice the rate of other vehicle types. Furthermore, recent data indicates that the accident rate per mile traveled for taxis is approximately 4.7 times higher than for trucks and 12.2 times higher than for buses^8)^, highlighting a substantial disparity in safety outcomes among commercial vehicles. Despite these concerning statistics, no previous study has investigated the effects of in-vehicle CO2 concentration on taxi drivers’ cognitive behavior, underscoring the social importance and novelty of this research.

## Methods

### Participants

Eight male taxi drivers (mean age 49.3; standard deviation [SD], 15.8; range, 28–72 years) participated in this preliminary study. All participants held Class II driver’s licenses and worked regular 24-hour shifts at least twice weekly. The sample size was similar to a recent driving simulator pilot study^9)^, considering both its methodological approach and practical constraints regarding professional driver availability.

Recruitment was conducted through a single cooperative taxi operator, with no specific criteria for age or driving experience to capture a representative cross-section of the taxi driver population.

### Ethical considerations

The study was approved by the Ethics Committee at the National University Corporation of Electro-Communications (approval number H23104). Written informed consent was obtained from all participants after receiving detailed explanations of the study procedures.

### Apparatus and experimental environment

The driving simulation was conducted using Euro Truck Simulator 2 software (SCS Software, Prague, Czech Republic), modified with the “Project Japan” add-on to accurately represent the Japanese road environment. The simulation was presented on a 27-inch display with 2,560 x 1,440 pixel resolution and a 144 Hz refresh rate. Participants operated the simulation using a Logitech G29 steering wheel and pedal set. The simulated vehicle was a right-hand drive Ford Explorer Platinum 2019 (version 1.01.47; Ford Motor Company, Dearborn, MI, USA) with automatic transmission. The simulated driving route was designed to represent roads in the Shikoku and Kansai regions of Japan. The 60-minute route began in an urban area of Kagawa Prefecture and included a mix of expressways and ordinary roads, featuring notable landmarks, such as the Akashi Kaikyo Bridge. While the simulated distances were scaled to 1/19th of actual distances, road widths were reproduced at full scale to maintain realistic driving conditions. To simulate typical driving conditions, the simulation time was set to 10:00 a.m., with traffic and other vehicle behaviors set to default parameters without extraordinary events, such as severe congestion or hazardous interruptions.

The experiment was conducted in a temperature-controlled room (26°C) under two CO2 conditions: LOW (≤500 ppm) and HIGH (5,000 ppm ± 10%). The HIGH condition was achieved using sublimating dry ice with controlled ventilation. CO2 levels were continuously monitored using a T&D TR-76Ui-S data logger (T&D Corporation, Matsumoto, Japan) (accuracy: ± [50 ppm + 5% of reading]), centrally located at 1.5 m height.

### Experimental design and procedure

A within-subjects design was employed with counterbalanced HIGH and LOW CO2 conditions, separated by a 1-week interval. Each session consisted of a 30-minute acclimation period under the designated CO2 condition, followed by a 15-minute practice drive, a brief rest, and the 1-hour main experimental task. Participants were blinded to the CO2 conditions.

### Measures

Three complementary measures were used to assess driving performance:

1. Two-back task: Participants responded verbally to audibly presented single-digit numbers at 10-second intervals, indicating matches with numbers presented two steps earlier. This task evaluated working memory and attention maintenance during driving. The two-back task produced two performance indicators: the incorrect response rate (NG: “No Good”) and the no-response rate (NA: “No Answer”).

2. LED response task: An LED in the lower right corner of the display was randomly illuminated at 10–20 second intervals. Participants pressed a button to extinguish the light, with responses exceeding 2 seconds were recorded as errors.

3. Driving skill evaluation: An experienced driving instructor with 17 years of license testing experience assessed performance using standard Japanese driving license test criteria^10)^. The evaluation covered four major categories: vehicle control (steering stability, acceleration/deceleration), rule compliance (signal observation, lane discipline), safety awareness (intersection behavior, following distance), and technical skills (parking, lane changing). The evaluator, blinded to CO2 conditions, recorded point deductions across 65 standardized assessment items, focusing on frequency rather than the standard pass/fail thresholds (70 points for regular licenses, 80 for Type 2).

### Data analysis

Analyses were conducted using Python 3.9 (Python Software Foundation) with Pandas and statsmodels libraries. Generalized linear mixed models (GLMM) analyzed the two-back task (incorrect and non-response rates) and LED response task (reaction time), while Poisson regression analyzed error counts and driving skill evaluations. All models included CO2 concentration as a fixed effect and participant ID as a random effect. Statistical significance was set at p<0.05, with effect sizes calculated using Cohen’s d. A supplementary correlation analysis examined individual differences in performance measures between conditions.

## Results

### Two-back task performance

**Fig. 1:**
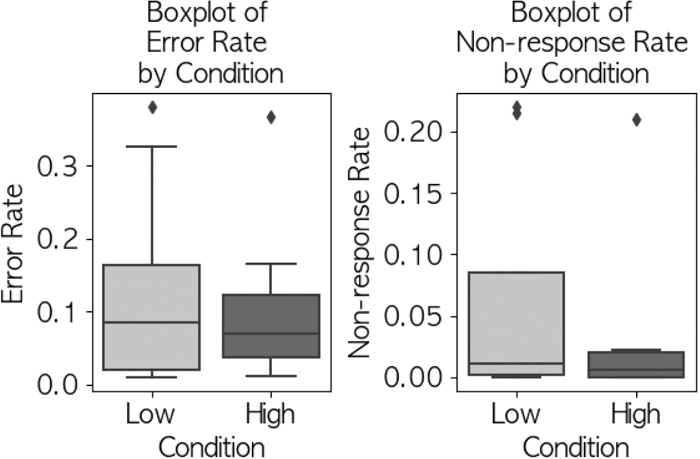
Incorrect and no-response rates for two-back tasks by condition

Two-back task performance showed minimal differences between conditions. Mean incorrect response rates were similar (HIGH: 0.091, LOW: 0.095), as were non-response rates (HIGH: 0.032, LOW: 0.034). GLMM analysis revealed no significant effect of CO2 concentration (NG rate: p=0.733; NA rate: p=0.485) with small effect sizes (Cohen’s d=0.15 and 0.13 respectively). Figure 1 illustrates these results.

### LED response task

**Fig. 2:**
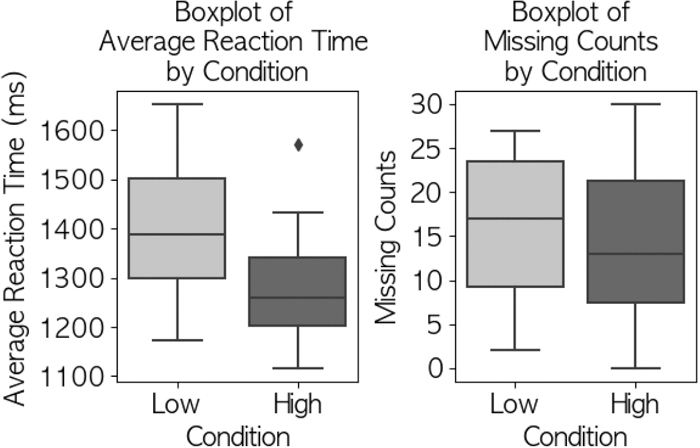
RTs and number of errors for LED assignments by condition

GLMM analysis revealed significantly faster reaction times in the HIGH CO2 condition (mean 1,440.87; SD, 78.34 s) compared to the LOW condition (mean 1,553.92; SD, 83.45 s; p<0.001, β=113.034, SE=26.658, 95% CI, 60.785–165.282). The effect size was moderate (Cohen’s d=0.72). Error rates showed no significant difference between conditions (HIGH: mean 14.3; SD, 5.4; LOW: mean 16.8; SD, 6.2; p=0.479, Cohen’s d=0.43). Figure 2 illustrates these results.

### Driving skill evaluation

**Fig. 3:**
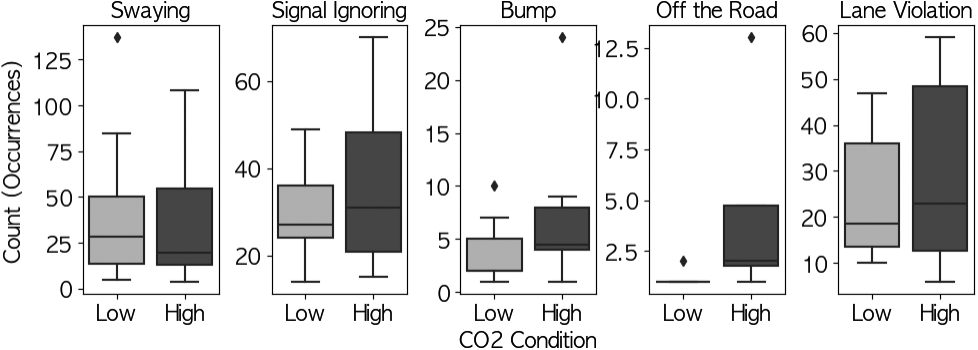
Number of point reductions by condition for items for which the increase in the number of point reductions was significant or tended to be significant.

Poisson regression analysis, interpreted through the Skill-Rule-Knowledge (SRK) model^14)^ and multiple resource theory^11)^, revealed distinct patterns in driving performance degradation. In the HIGH CO2 condition, participants exhibited significantly more frequent occurrences of wobbling (β=0.1595; standard error [SE], 0.079; 95% CI, 0.004–0.315; p=0.044; Cohen’s d=0.52), failure to signal (β=0.35; SE, 0.17; 95% CI, 0.03–0.66; p=0.045; Cohen’s d=0.44), contact with other vehicles or objects (β=0.7085; SE, 0.241; 95% CI, 0.235–1.182; p=0.003; Cohen’s d=0.60), and wheel excursions (β=1.3218; SE, 0.471; 95% CI, 0.398–2.246; p=0.005; Cohen’s d=0.47). These effects particularly impacted skill-based behaviors that typically operate with minimal conscious control, while showing less influence on rule-based behaviors such as traffic zone adherence (β=−0.1772; SE, 0.097; 95% CI, −0.367 to 0.013; p=0.067; Cohen’s d=0.40), as illustrated in Figure 3. This pattern aligns with multiple resource theory’s prediction of selective resource depletion, where visual-spatial processing and motor control precision show greater sensitivity to CO2 effects, as demonstrated in recent studies^16,17)^. Research has observed that elevated CO2 levels (0.5%) in a spaceflight analog environment specifically impaired visual-motor tasks while having less impact on verbal working memory tasks^16)^, supporting the differential vulnerability of cognitive functions to CO2 exposure.

Correlation analysis examined whether individual performance patterns were maintained across CO2 conditions (eg, whether participants who performed well in LOW condition also performed well in HIGH condition). No significant correlations were found between conditions for any performance measures, suggesting that individual differences had little systematic influence on how participants responded to increased CO2 levels.

## Discussion

### Two-back task performance

The two-back task showed no significant effects of CO2 concentration on performance (NG rate: β=0.0913; SE, 0.129; 95% CI, −0.161 to 0.344; p=0.733; NA rate: β=−0.0772; SE, 0.097; 95% CI, −0.367 to 0.013; p=0.485), with small effect sizes (Cohen’s d: 0.15 for incorrect answers, 0.13 for non-responses), contrasting with previous studies reporting cognitive declines under elevated CO2^5,6)^. This discrepancy can be analyzed through three key factors: First, task complexity differences — our focused working memory task may be less sensitive than the complex decision-making assessments used in previous studies. This is supported by previous research^4)^, which found that performance on complex decision-making tasks decreased significantly at moderate CO2 levels while simpler cognitive functions remained relatively unaffected. Other studies^18)^ further demonstrated that CO2 exposure differentially affects cognitive domains, with higher-order executive functions showing greater vulnerability than basic attentional processes or working memory.

Second, exposure duration variations — our 1-hour protocol versus typical 2.5+ hour studies may have limited cumulative CO2 effects. Research has demonstrated that cognitive performance decrements were more pronounced after extended CO2 exposure (>2 hours) compared to shorter exposures^18)^. Other studies^17)^ also reported time-dependent cognitive responses to elevated CO2, with adaptation processes beginning to appear after approximately 90 minutes of exposure.

Third, participant expertise — taxi drivers may have developed compensatory mechanisms through regular exposure to elevated CO2 levels. This pattern of preserved working memory performance, when contrasted with significant impairments in driving tasks, suggests differential vulnerability of cognitive functions to CO2 exposure, aligning with multiple resource theory’s^11)^ prediction that verbal working memory may be more resilient than visual-spatial processing and motor control functions.

### LED response task performance

While reaction times were significantly faster in the HIGH CO2 condition (mean 1,440.87; SD, 78.34 ms) compared to the LOW condition (mean 1,553.92; SD, 83.45 ms; β=113.034; SE, 26.658; 95% CI, 60.785–165.282; p<0.001; GLMM analysis with CO2 condition as fixed effect and participant ID as random effect). The effect size was moderate (Cohen’s d=0.72). Error rates showed no significant difference between conditions (HIGH: mean 14.3; SD, 5.4; LOW: mean 16.8; SD, 6.2; p=0.479, Cohen’s d=0.43). This finding warrants careful interpretation through multiple theoretical frameworks of attention and cognitive resources. The multiple resource theory^11)^ posits that individuals strategically allocate cognitive resources when performing concurrent tasks, particularly under stress conditions, while the compensatory control model^12)^ explains how individuals adapt their performance under cognitive constraints. In our study context, these frameworks suggest that elevated CO2 may have led drivers to unconsciously prioritize the simpler LED task over more complex driving tasks, as it was less susceptible to carryover effects from previous trials and required fewer cognitive resources. The absence of significant differences in error rates (Cohen’s d=0.43) suggests a speed-accuracy trade-off^13)^, where faster responses often come at the cost of reduced accuracy. This trade-off pattern, combined with the performance changes observed in driving tasks, indicates an adaptive strategy to optimize limited cognitive resources rather than enhanced performance. The faster reaction times might thus represent a compensatory mechanism where attention is redirected to simpler tasks when overall cognitive capacity is compromised by high CO2 levels.

### Driving skill evaluation

The increased driving errors under HIGH CO2 conditions can be interpreted through both the SRK model^14)^ and multiple resource theory^11)^. The SRK model suggests that elevated CO2 particularly affected automated skill-based behaviors (wobble, contact, wheel excursion, and signaling), which are typically performed with minimal conscious attention as they are highly automated actions requiring little cognitive effort when mastered. It should be emphasized that all participants remained fully conscious throughout the study; the observed effects represent subtle performance decrements in automated behaviors rather than any changes in consciousness levels.

The multiple resource theory provides additional insight, suggesting that CO2 may differentially impact specific attentional resources, particularly those associated with visual attention and motor control. This interpretation is supported by the pattern of results across tasks: impairment of automated driving behaviors, faster but potentially compensatory LED task performance, and preserved verbal working memory in the two-back task. This pattern suggests strategic reallocation of attentional resources under CO2-induced cognitive constraints.

### Methodological insights and future directions

This preliminary study demonstrates the feasibility of investigating CO2 effects on driving performance and provides key methodological insights. The experimental protocol refinement efforts demonstrated several key findings. The two-session design with 1-week intervals proved feasible for professional drivers, while the 60-minute driving task duration was sufficient to observe meaningful performance changes. Additionally, the combined use of multiple performance measures, including the two-back task, LED response, and driving evaluation, provided complementary insights into different aspects of driver performance.

Measurement optimization emerged as another crucial methodological outcome. The driving evaluation criteria adapted from license testing showed particular sensitivity to CO2 effects, suggesting its utility for future studies. Based on observed effect sizes (Cohen’s d: 0.13–0.72), we estimate a minimum sample size of 40 participants for future studies. Moreover, specific behavioral measures showing the largest effects, such as wheel excursions, should be prioritized in future research to maximize statistical sensitivity.

Future research should address current limitations through: (1) increased sample size and diversity, including female drivers and varied experience levels; (2) multiple CO2 concentration levels for establishing dose-response relationships and varied exposure durations to examine temporal effects; (3) implementation of physiological measures such as EEG and heart rate variability to understand underlying mechanisms and analyze performance in terms of the Yerkes-Dodson law^15)^, which relates arousal level to performance; and (4) validation through on-road studies using vehicles equipped with CO2 sensors, digital tachographs, and drive recorders. The observed sensitivity of skill-based driving behaviors to CO2 exposure and the potential role of attentional resource allocation suggest these measures should be prioritized in future investigations.

### Considerations for social implementation

Our findings highlight two critical areas for managing in-vehicle CO2 concentrations. First, ventilation systems require improvement through the development of adaptive control systems that monitor and maintain appropriate air quality. Such systems could incorporate real-time CO2 monitoring sensors or predictive algorithms based on ventilation mode, occupancy, and driving conditions.

Second, driver education programs should be implemented, particularly for commercial drivers operating vehicles with multiple occupants or during extended periods. This is especially crucial for taxi, bus, and truck operators, who often use internal air circulation mode during adverse weather or in regions with air pollution or pollen allergy concerns. Since human breath contains high concentrations of CO2 (20,000 to 30,000 ppm), CO2 levels may be particularly high when express buses and taxis are loaded with multiple passengers and operate in the internal air circulation mode for long periods. Special consideration should be given to situations involving CO2-generating cargo, such as fruit trees that emit CO2 as they ripen (eg, apples, bananas) and trucks loaded with dry ice for transporting frozen goods, medicines, specimens, and bodies (except those with spatially separated cargo compartments).

A promising alternative approach would be the implementation of advanced outside air intake systems with high-efficiency filtration that can provide clean air while avoiding the CO2 accumulation associated with internal air recirculation. Studies^19)^ have demonstrated that fractional recirculation systems, which combine partial recirculation with filtered outside air intake, can simultaneously reduce both particulate matter and CO2 concentrations in vehicle cabins. Similarly, recent advances in filtration technology could enable clean outside air introduction even in areas with air pollution or allergen concerns, potentially eliminating the need to choose between poor air quality and elevated CO2 levels.

While the Japanese automotive industry has addressed cabin air quality through initiatives like Japan Automobile Manufacturers Association’s volatile organic compounds reduction program, no specific standards exist for CO2 concentrations. Given the potential impact on traffic safety, establishing appropriate standards and regulations for in-vehicle CO2 management should be considered.

### Future research directions

Building on our methodological findings, future research should emphasize real-world validation through studies using actual vehicles equipped with CO2 sensors and drive recorders. Intervention studies evaluating the effectiveness of CO2 reduction measures, such as improved ventilation systems and driver feedback mechanisms, will be crucial for practical implementation. Additionally, investigating the relationship between CO2 exposure and different types of attentional resources will enhance our understanding of the cognitive adaptation processes observed in this study.

## Conclusion

This preliminary study demonstrates that elevated CO2 concentrations significantly affect driving performance in a simulated environment, particularly impacting skill-based behaviors, such as vehicle control stability, signaling, and lane maintenance. While working memory remained unaffected, the pattern of results suggests that CO2 exposure primarily influences automated driving behaviors, interpreted through Rasmussen’s SRK model and multiple resource theory.

The findings highlight the critical importance of managing in-vehicle CO2 concentrations for driving safety and provide a methodological foundation for future research. Effect sizes observed across performance measures suggest the need for larger-scale studies with diverse driver populations, physiological measurements, and real-world driving conditions to further understand how CO2 levels impact driving performance and develop effective countermeasures.
